# Neuroimaging evidence of deficient axon myelination in Wolfram syndrome

**DOI:** 10.1038/srep21167

**Published:** 2016-02-18

**Authors:** Heather M. Lugar, Jonathan M. Koller, Jerrel Rutlin, Bess A. Marshall, Kohsuke Kanekura, Fumihiko Urano, Allison N. Bischoff, Joshua S. Shimony, Tamara Hershey, the Washington University Wolfram Syndrome Research Study Group, P. Austin, B. Beato, E. Bihun, T. Doty, G. Earhart, S. Eisenstein, J. Hoekel, R. Karzon, A. Licis, L. Manwaring, A. R. Paciorkowski, Y. Pepino de Gruev, A. Permutt, K. Pickett, S. Ranck, A. Reiersen, L. Tychsen, A. Viehoever, J. Wasson, N. H. White

**Affiliations:** 1 Department of Psychiatry, Washington University School of Medicine, St. Louis, MO, USA; 2 Department of Neurology, Washington University School of Medicine, St. Louis, MO, USA; 3 Mallinckrodt Institute of Radiology, Washington University School of Medicine, St. Louis, MO, USA; 4 Department of Pediatrics, Washington University School of Medicine, St. Louis, MO, USA; 5 Department of Cell Biology, Washington University School of Medicine, St. Louis, MO, USA; 6 Department of Medicine, Washington University School of Medicine, St. Louis, MO, USA; 7 St. Louis Children’s Hospital, Washington University School of Medicine, St. Louis, MO, USA; 8 Department of Surgery, Washington University School of Medicine, St. Louis, MO, USA; 9 Program in Physical Therapy, Washington University School of Medicine, St. Louis, MO, USA; 10 Department of Ophthalmology, Washington University School of Medicine, St. Louis, MO, USA; 11 Program in Audiology and Communication Sciences, Washington University School of Medicine, St. Louis, MO, USA; 12 Department of Neurology, University of Rochester Medical Center, NY, USA; 13 Department of Physical Therapy, University of Wisconsin, WI, USA; 14 Department of Neurology, University of California San Francisco, CA, USA

## Abstract

Wolfram syndrome is a rare autosomal recessive genetic disease characterized by insulin dependent diabetes and vision, hearing and brain abnormalities which generally emerge in childhood. Mutations in the *WFS1* gene predispose cells to endoplasmic reticulum stress-mediated apoptosis and may induce myelin degradation in neuronal cell models. However, *in vivo* evidence of this phenomenon in humans is lacking. White matter microstructure and regional volumes were measured using magnetic resonance imaging in children and young adults with Wolfram syndrome (n = 21) and healthy and diabetic controls (n = 50). Wolfram patients had lower fractional anisotropy and higher radial diffusivity in major white matter tracts and lower volume in the basilar (ventral) pons, cerebellar white matter and visual cortex. Correlations were found between key brain findings and overall neurological symptoms. This pattern of findings suggests that reduction in myelin is a primary neuropathological feature of Wolfram syndrome. Endoplasmic reticulum stress-related dysfunction in Wolfram syndrome may interact with the development of myelin or promote degeneration of myelin during the progression of the disease. These measures may provide objective indices of Wolfram syndrome pathophysiology that will be useful in unraveling the underlying mechanisms and in testing the impact of treatments on the brain.

Wolfram syndrome (OMIM #222300) is a rare autosomal recessive genetic disease originally described as the combination of insulin dependent diabetes mellitus, optic nerve atrophy, diabetes insipidus and deafness^1^. Neurodegeneration and neurological features were thought to appear at later stages of the disease, ultimately leading to death in middle adulthood^2^. Since the major causative gene (*WFS1*)^3^ has been identified, the known phenotype of Wolfram syndrome has expanded. It is now evident that not all of these symptoms are present in all of the patients who are genetically identified^4^,^5^,^6^ and that neurologic abnormality is present even at early stages of the disease^7^,^8^,^9^.

Mutations in the *WFS1* gene appear to predispose insulin producing pancreatic β-cells to endoplasmic reticulum (ER) stress-mediated apoptosis via disruption of cellular calcium homeostasis^10^,^11^,^12^,^13^,^14^,^15^,^16^ and this mechanism also accounts for central nervous system degeneration, including cellular evidence of myelin degradation^17^. However, it is not fully known how/if this presumed cellular pathophysiology manifests at the systems level in the human brain or interacts with neurodevelopmental processes. For example, myelinating cells are highly sensitive to ER stress, and the pathogenesis of a number of neurodevelopmental myelin disorders has ER stress dysfunction as a root cause^18^. Thus, it is possible that Wolfram syndrome may preferentially affect the myelination of axons during brain development, and that this pathology underlies many of the neurologic symptoms observed. Rare neuropathological examinations of Wolfram patients’ brains have noted loss of myelinated axons throughout the visual system, grossly smaller brainstem (particularly ventral pons) and less white matter in the cerebellum^19^. In a previous small neuroimaging study of Wolfram patients (n = 11), we found lower white and gray matter regional volumes and abnormalities in white matter microstructure compared to convenience controls^9^. However, these findings were limited by a small sample size, lack of explicitly matched control groups, and lack of assessment of neurologic symptoms. Thus the potential preferential vulnerability of white vs. gray matter, and myelin vs. axons to Wolfram syndrome and the relationship of these neuropathologies to neurological symptoms have not been determined.

Given that the neurological features of Wolfram syndrome are life-threatening, evolve over development, and are important targets of intervention, these aspects require greater attention. While molecular and cellular experiments have led the field closer to identifying potential interventions for degenerative processes in Wolfram syndrome, the identification of reliable neurological biomarkers, their underlying neuropathophysiology, and clinical correlates is in its infancy. Thus, the goals of the current study were to better define the pattern of selective neurologic vulnerability and determine whether *in vivo* brain measurements, particularly of myelination, relate to neurologic symptoms. By comparing a sample of Wolfram patients (n = 21) to age and gender equivalent control groups with identical structural neuroimaging and behavioral measures, we can ask these questions in a powerful and controlled manner.

## Materials and Methods

### Participants

The Human Research Protection Office at Washington University in Saint Louis approved the study and methods were carried out in accordance with the approved guidelines. Written consent was obtained by all participants prior to any testing. For children under the age of 18, written consent was obtained by parents or guardians, and the children assented to participation in the study. Patients with Wolfram syndrome were recruited primarily through the Washington University Wolfram Syndrome Registry (http://wolframsyndrome.dom.wustl.edu/) to participate in a longitudinal natural history study (Washington University Wolfram Syndrome Research Clinic). When enrolled, patients were under the age of 30, aware of their diagnosis, and genetically confirmed to have a *WFS1* mutation. Patients were annually evaluated by physician specialists and underwent neuropsychological testing and magnetic resonance imaging (MRI). Some of these data have been previously reported^5^,^7^,^8^,^9^,^20^,^21^,^22^,^23^. To maximize the sample size for this analysis, we pooled patients evaluated in 2012 (n = 1), 2013 (n = 16) and 2014 (n = 4) for a total of 21 WFS patients, ranging in age from 6–26 years (67% Caucasian, Non-Hispanic). Participants whose MRIs were previously reported (n = 11) are part of this sample, but their data in this analysis are from different years (2010 and 2011).

The age and gender equivalent comparison group consisted of 24 individuals with Type 1 diabetes mellitus, ranging in age from 7–26 years (96% Caucasian, Non-Hispanic), and 26 non-diabetic healthy controls ranging in age from 6–26 years (79% Caucasian, Non-Hispanic). Diabetic individuals were recruited from the Pediatric Diabetes Clinic at Saint Louis Children’s Hospital and Washington University School of Medicine in Saint Louis, and healthy controls were either recruited from the community or were healthy siblings of the diabetic participants. Controls were excluded for self-reported neurological or psychiatric diagnoses, use of psychoactive medication, premature birth (<36 weeks gestation) or other complications, and contraindications for MRI.

### Assessments

Wolfram patients and controls underwent MRI scans, cognitive, smell, balance, and limited gait assessment over the course of 1–4 days. Wolfram patients underwent further clinical testing in neurology, ophthalmology, and audiology.

#### Testing in all participants

*MRI Acquisition:* Prior to MRI scans, all participants were confirmed to have blood glucose levels between 70 and 300mg/dl. For each participant, the following scans were acquired on the same Siemens 3 Tesla Tim Trio at Washington University: *T1-weighted Magnetization-Prepared Rapid Gradient-Echo (MPRAGE) sequence*: Sagittal acquisition, repetition time (TR) = 2400, echo time (TE) = 3.16, inversion time (TI) = 1000, voxel resolution = 1 × 1 × 1mm, Time = 8:09 min. *T2-weighted MR:* Sagittal acquisition, TR = 3200, TE = 455, voxel resolution = 1 × 1 × 1mm, Time = 4:43 min. *T2 fluid attenuated inversion recovery (FLAIR):* Transverse acquisition, TR = 9190, TE = 98, TI = 2500, voxel resolution = 0.86 × 0.86 × 3 mm, Time = 3:59 min. *Diffusion Tensor Imaging (DTI):* The echo planar sequence consisted of 27 directions with b-values ranging from 0 to 1400 s/mm^2^. Transverse acquisition, TR = 12300, TE = 108, voxel resolution = 2 × 2 × 2 mm, Time = 5:44 min. *Behavioral Measure*s: Glycated hemoglobin (Hb_A1c_) was collected from all participants as an index of average plasma glucose concentration over the past 2–3 months. Prior to cognitive testing, all participants were confirmed to have blood glucose levels between 70 and 300mg/dl. A verbal intelligence quotient (VIQ) was calculated using the Vocabulary and Similarities subtests of the Wechsler Abbreviated Scale of Intelligence^24^. Verbal intelligence of a participant’s parent was assessed using the Wechsler Test of Adult Reading (WTAR)^25^. Smell identification was tested with the University of Pennsylvania’s Smell Identification Test (UPSIT)^26^. The mini-Balance Evaluation Systems Test (mini-BESTest) was used to rate overall balance^8^,^27^. Two subscores of the mini-BESTest were used to measure gait (Timed Get Up and Go or *TUG*, and TUG with Dual Task, or *DT-TUG*).

#### Testing in Wolfram patients only

The Wolfram Unified Rating Scale (WURS), designed to measure the severity of symptoms commonly associated with Wolfram syndrome^21^ and the Physical and Neurological Examination for Subtle Signs (PANESS), an age-normalized clinical assessment tool used to evaluate gross motor function^28^,^29^, were performed by a neurologist. Patients were tested for color vision (total score on the Hardy-Rand-Rittler, performed under a MacBeth Easel lamp), best-corrected visual acuity (logmar score for both eyes on a Snellan optotype), and retinal nerve fiber layer thickness (averaged across eyes on the Zeiss Cirrus high density optical coherence tomography, HD-OCT, 4000–5444 version 4.5.1.11; CarlZeiss Meditec Inc, Dublin, CA)^22^. Also, patients were tested for high frequency hearing, pure tone hearing and speech intelligibility (Madsen Orbiter-922 audiometer, Audioscan Verifit)^23^. Finally, myelin basic protein levels were measured in serum. Blood samples were initially collected in ethylenediaminetetraacetic acid-containing blood collection tubes and centrifuged at 10,000 g for 5 min. Supernatant was aliquoted and immediately frozen at −80 °C until later analysis. Myelin basic protein levels in ng/ml were determined using an enzyme-linked immunosorbant assay (ELISA) kit (R&D System, Minneapolis, MN).

### Neuroimaging analyses

#### Head size

*Skull circumference:* Using custom code, we measured skull circumference using the MPRAGE, at a resolution of 1 mm^3^, and the T2 image. Scans were registered to atlas space with affine rigid body rotations but no stretch, assuring that the slice of the anterior commissure-posterior commissure (AC-PC) line was consistently oriented across subjects without altering the size of the brain. Scans were then processed using the Brain Extraction Tool (BET) within the Oxford Centre for Functional MRI Software Library (FSL)^30^,^31^ to create a binary mask at the outer boundary of the skull with settings individually optimized. The binary masked slice at the AC-PC line was processed by finding a start voxel at the edge of the brain and then tracing the periphery of the masked brain until returning to the starting point. The circumference was computed by accumulating the distance steps between adjacent voxels along the periphery of the brain mask. *Estimate of total intracranial volume* (*eTIV):* Freesurfer (v5.3) was used to reconstruct the brain from volumetric and surface based registration to an atlas^32^. The one-parameter scaling factor that was applied to each individual atlas registration was used as an estimate of total intracranial volume as previously validated^33^.

#### Global brain variables

Total cortical gray matter volume, total cortical white matter volume, average surface area and average thickness were extracted from Freesurfer output for analyses.

#### Subcortical region volumes

Freesurfer was used to extract regional gray and white matter brain volumes from anatomically defined regions (brainstem, cerebellar gray matter, cerebellar white matter, thalamus, pallidum, corpus callosum, hippocampus, amygdala, caudate, putamen, and accumbens). Regions were averaged between right and left hemispheres as appropriate and corrected for eTIV.

The brainstem was manually segmented into its major components: midbrain, basilar (ventral) pons, tegmentum (dorsal pons), and medulla. The atlas was rotated to align the brainstem vertically, and individual MRI images were aligned to this template^9^. Four borders were then manually defined in 3D Slicer (http://www.slicer.org)^34^. Intra-class correlations for two independent raters and test re-test by a single rater, on a portion of the sample, were high for all four borders (>0.98).

#### A priori cortical regions

For surface-based cortical metrics, cortical maps were generated in Freesurfer by identifying the gray/white matter border and pial surfaces in each individual and then applying a triangular tessellation to the cortical surface^35^. Three types of surface based measurements were then calculated at each vertex of the triangular mesh: cortical thickness (the distance between the white and pial surface), surface area (the sum of the areas of the triangles connected to a vertex) and gray matter volume (the product of cortical thickness and surface area).

Due to the visual and auditory impairment associated with Wolfram syndrome, volume, area and thickness in primary and secondary visual (V1 and V2) and primary and secondary auditory cortices were selected a priori as regions of interest. Regions were averaged between right and left hemispheres. Volumes and surface area were corrected for eTIV, and thicknesses were corrected for global thickness.

#### Vertex-wise cortical metrics (Query, Design, Estimate, Contrast; QDEC)

Cortical volume, surface area, and thickness were also explored in a landmark-independent, vertex by vertex method, using Freesurfer’s group analysis tool, QDEC. Data were smoothed using a full width/half-maximum Gaussian kernel of 15 mm for thickness and 10 mm for area and volume.

#### White matter tracts

DTI scans were skull stripped using the FSL Brain Extraction Tool and then registered to atlas and corrected for eddy current distortion effects using the FSL Diffusion Toolkit (FDT)^30^. To ensure that motion artifact was not responsible for any findings observed in the DTI data, outlier DTI data was collected from the image processing steps, and the number of rejected outlier encodes per subject was calculated. In order to estimate white matter connectivity for individually defined tracts, both seed and waypoint masks were created and defined on the Montreal Neurological Institute atlas (MNI152) brain. Each connectivity map was then thresholded at 1% to remove extraneous pathways and converted into binary masks for the purpose of extracting mean fractional anisotropy (FA), axial diffusivity (AD), and radial diffusivity (RD) in major white matter tracts (corticospinal, optic radiations, middle cerebral peduncle, inferior fronto-occipital fasciculus, arcuate fasciculus, uncinate fasciculus, acoustic radiations, corpus callosum), as described^9^,^36^.

#### Voxel-wise white matter (TBSS)

Tract-based spatial statistics (TBSS) was used to perform voxel-wise analyses of all white matter tracts, as previously described^9^,^37^. FA, AD, and RD images were calculated (FDT). FA images were projected onto the mean FA skeleton, which represents the center of white matter tracts, and thresholded at FA = 0.2.

### Statistical analyses

Healthy control and diabetic control groups were combined to simplify the statistical models and maximize power. Previous analyses have not found differences between these two control groups on any MRI outcome measures^9^. To compare groups on clinical, behavioral, whole brain, regional and tract measures, we performed univariate analyses covarying age and gender using SPSS^©^ Version 22. We also explored whether there was an age x group interaction on the brain variables that differed between groups using hierarchical linear regression. For vertex-wise cortical metrics (QDEC), groups were compared using general linear models for each hemisphere. Additional covariates were added for each analysis to avoid multicollinearity (thickness: gray matter volume and area; area: gray matter volume, thickness, and cortical white matter volume; volume: thickness and area). Multiple comparison corrections were applied using the Monte Carlo permutation cluster analyses. For voxel-wise DTI parameter analyses (TBSS), groups were compared using general linear models, covarying age and gender. Multiple comparison corrections were applied using a permutation-based statistical approach within Randomize^38^. Brain and behavioral measures which had significant group effects or were abnormal compared to clinical norms were selected for further correlational analysis within the Wolfram group, controlling for age and gender. Significance was set at p < 0.05.

## Results

### Participants

Twenty-one Wolfram patients and 50 age and gender equivalent controls were assessed. Of the 21 Wolfram patients examined, all had optic atrophy, 20 had insulin dependent diabetes mellitus, 14 had diabetes insipidus, and 10 had hearing loss. Age of diagnosis with each Wolfram syndrome characteristic and genetic mutation for each patient, as well as age at study, is reported in Supplementary Table S1 with siblings noted. The Wolfram group did not differ from the combined control group in age (F_1,69_ = 0.34, p = 0.859), gender distribution (χ^2^ (N = 71) = 0.59, p = 0.444) or parental estimated verbal IQ (WTAR, F_1,50_ = 1.52, p = 0.224), even when considering subsamples due to missing data. The Wolfram group had lower Hb_A1c_ than the type 1 diabetic group (F_1,41_ = 9.02, p = 0.005) but these groups did not differ in diabetes duration (F_1,42_ = 0.39, p = 0.536) or blood glucose levels pre or post-MRI (F_1,42_ = 0.01, p = 0.905; F_1,40_ = 1.04, p = 0.313) (Table 1).

Some variables had missing data. Six healthy controls, four diabetic controls, and seven Wolfram patients did not have parental WTAR data. Similarly, eight Wolfram patients were missing a verbal IQ score (primarily due to being non-native English speakers). Hb_A1c_ was obtained by all participants except for one healthy control. Diabetes duration and pre-MRI blood glucose levels were obtained on all diabetic participants, but two Wolfram patients were missing post-MRI blood glucose levels. With regard to clinical data collected in the Wolfram group only, one Wolfram patient did not complete the WURS, three did not complete the gait assessment for double support, three did not have retinal thickness measures, four did not have speech intelligibility measures, and one was missing myelin basic protein levels. Due to a cortical brain anomaly, one Wolfram patient was excluded from all neuroimaging analyses except for carefully inspected brainstem segmentation volumes, and two healthy controls had MPRAGE but no DTI data.

### Motor, sensory and clinical variables

Wolfram and control groups did not differ in verbal intelligence (VIQ, F_1,59_ = 0.58, p = 0.449) or the *DT-TUG* portion of the mini-BESTest (F_1,67_ = 2.00, p = 0.162). However, the Wolfram group performed worse than controls on smell identification (UPSIT, F_1,67_ = 19.67, p < 0.001), the mini-BESTest (summary score, F_1,67_ = 42.74, p < 0.001), and the *TUG* task portion of the mini-BESTest (F_1,67_ = 9.02, p = 0.004) (Table 1). Clinical variables assessed only in Wolfram patients were abnormal compared to normative clinical data (Table 2).

### Neuroimaging

#### Head size

The Wolfram group had smaller skull circumference (F_1,66_ = 5.81, p = 0.019) and eTIV (F_1,66_ = 6.48, p = 0.013) compared to controls (Table 3). Importantly, all other brain volume measures analyzed below were corrected for eTIV. Interestingly, Wolfram patients were also shorter than controls (F_1,66_ = 6.0, p = 0.017). eTIV, height and skull circumference were all highly correlated with each other within Wolfram and within controls (p < 0.001), with the exception of eTIV and height in the Wolfram group (r_20_ = 0.16, p = 0.493).

#### Global brain variables

The Wolfram group had lower total cortical white matter volume (F_1,66_ = 4.12, p = 0.046) and total subcortical gray matter volume (F_1,66_ = 12.00, p = 0.001) but greater average surface area (F_1,66_ = 4.52, p = 0.037) compared to controls. Groups did not differ in total cortical gray matter volume (F_1,66_ = 0.18, p = 0.676) or average cortical thickness (F_1,66_ = 2.28, p = 0.136) (Table 3).

#### Subcortical region volumes

The Wolfram group had lower volumes in the majority of subcortical regions (8/14) compared to controls after correcting for eTIV and covarying age and gender. These regions were: basilar (ventral) pons (F_1,67_ = 110.00, p < 0.001), tegmentum (dorsal) pons (F_1,67_ = 5.40, p = 0.023), midbrain (F_1,67_ = 4.56, p = 0.036), medulla (F_1,67_ = 7.13, p = 0.010), cerebellar white matter (F_1,66_ = 47.81, p < 0.001), cerebellar gray matter (F_1,66_ = 11.61, p = 0.001), thalamus (F_1,66_ = 27.99, p < 0.001), and pallidum (F_1,66_ = 15.16, p < 0.001). Only amygdala volume was greater in the Wolfram group compared to controls (F_1,66_ = 5.12, p = 0.027). There were no differences between groups in caudate (F_1,66_ = 0.62, p = 0.433), putamen (F_1,66_ = 3.59, p = 0.063), hippocampus (F_1,66_ = 0.04, p = 0.837), accumbens (F_1,66_ = 0.09, p = 0.769), or corpus callosum (F_1,66_ = 1.47, p = 0.230) volumes (Table 3).

#### A priori cortical regions

All four cortical regions of interest were different between groups in at least one cortical metric. For all metrics, the Wolfram group had lower values in the visual regions (V1 and V2) and higher values in the auditory regions (primary and secondary auditory cortices) than controls. A main effect of group was seen in V1 surface area (F_1,66_ = 8.95, p = 0.004), V1 gray matter volume (F_1,66_ = 17.83, p < 0.001), V1 thickness (F_1,66_ = 14.52, p =  < 0.001), V2 gray matter volume (F_1,66_ = 5.47, p = 0.022), V2 thickness (F_1,66_ = 11.53, p = 0.001), primary auditory cortex gray matter volume (F_1,66_ = 4.38, p = 0.040), secondary auditory cortex surface area (F_1,66_ = 18.22, p < 0.001), and secondary auditory cortex gray matter volume (F_1,66_ = 5.98, p = 0.017). There were no differences between groups in V2 surface area (F_1,66_ = 0.31, p = 0.582), primary auditory cortex surface area (F_1,66_ = 3.95, p = 0.057), primary auditory cortex thickness (F_1,66_ = 0.10, p = 0.749), or secondary auditory cortex thickness (F_1,66_ = 2.29, p = 0.135) (Table 4).

#### QDEC

**Figure 1:**
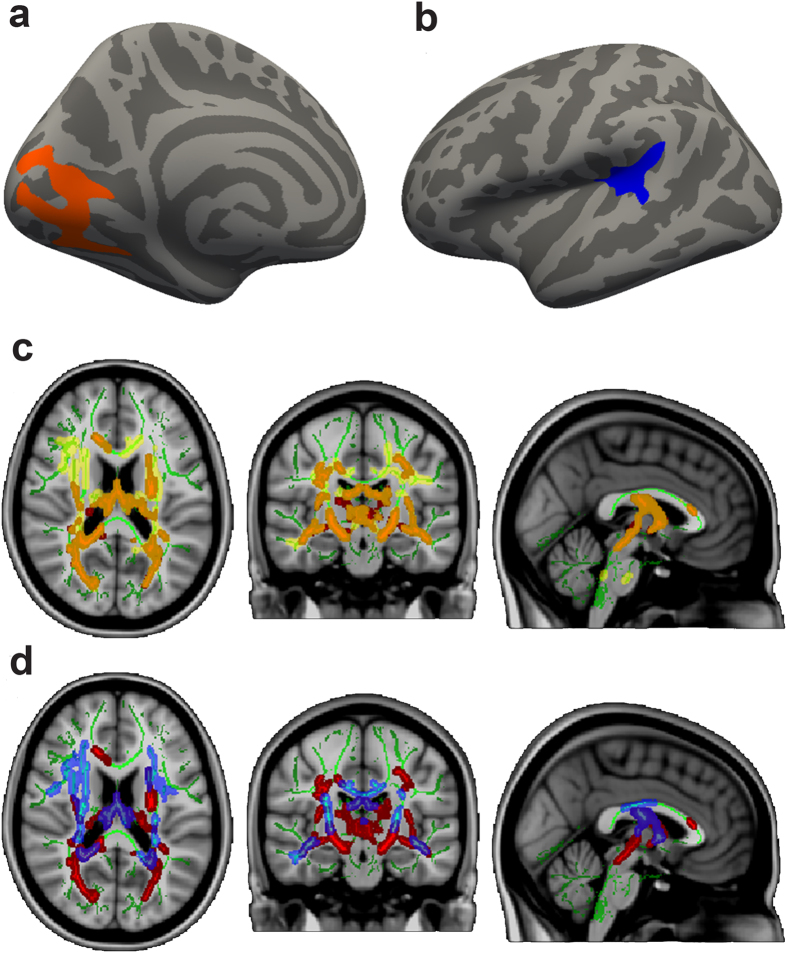
Differences in cortical thickness, volume and white matter microstructure between Wolfram and Control groups. (**a**) Cortical thickness was lower in the pericalcarine area in Wolfram Syndrome compared to Controls, after multiple comparison corrections, p = 0.0001. (**b**) Cortical volume was greater in the superior temporal area in Wolfram Syndrome compared to controls, after multiple comparison correction, p = 0.035. (**c**) Fractional anisotropy (FA, red) was lower, and radial diffusivity (RD, yellow) was higher compared to controls, after multiple comparison correction. Overlap between FA and RD differences are shown in orange. (**d**) Fractional anisotropy (FA, red) was lower, and axial diffusivity (AD, blue) was higher compared to controls, after multiple comparison correction. Overlap between FA and AD differences are shown in purple.

Vertex-wise cortical results were largely consistent with the a priori cortical findings. After correcting for multicollinearity and multiple comparisons, the Wolfram group had lower values in a number of pericalcarine regions in surface area (left, cluster size = 1729.09 mm^2^, cluster-wise p = 0.003), thickness (left, cluster size = 2225.27 mm, cluster-wise p < 0.001, Fig. 1a; right, cluster size = 896.93 mm, cluster-wise p = 0.007), and volume (left, cluster size = 2677.48 mm^3^, cluster-wise p < 0.001; right, cluster size = 1747.63 mm^3^, cluster-wise p < 0.001). In addition, the Wolfram group had lower volume in a parahippocampal region (right, cluster size = 781.79 mm^3^, cluster-wise p = 0.041). Conversely, the Wolfram group had higher values than controls in a number of regions in the temporal lobes, including surface area (right superiortemporal, cluster size = 1203.32 mm^2^, cluster-wise p = 0.041; left supramarginal, cluster size = 1555.03 mm^2^, cluster-wise p = 0.007) and volume (left superiortemporal, cluster size = 780.78 mm^3^, cluster-wise p = 0.035, Fig. 1b), as well as in the frontal lobe volume (right pars triangularis, cluster size = 919.75 mm^3^, cluster-wise p = 0.014) and thickness (right rostral middle frontal, cluster size = 997.65 mm, cluster-wise p = 0.004).

#### White matter tracts

Groups did not differ in the number of bad encodes due to movement (F_1,65_ = 1.01, p = 0.318)^39^. The Wolfram group had lower FA and higher RD in 5/8 white matter tracts and higher AD in 2/8 tracts compared to controls after co-varying age and gender. No differences were seen in the alternate directions (e.g. higher FA or lower RD and AD in Wolfram syndrome) (Table 5).

#### TBSS

Voxel-wise analyses of DTI parameters largely confirmed the tractography results. The Wolfram group had lower FA compared to controls across many white matter areas, including corticospinal tract, inferior fronto-occipital fasciculus, optic radiations, and parts of the corpus callosum body. The Wolfram group had higher RD in regions mostly overlapping with FA findings (middle cerebellar peduncle, corticospinal tract, inferior fronto-occipital fasciculus, optic radiations, inferior longitudinal fasciculus, and superior longitudinal fasciculus). The Wolfram group had higher AD than controls in more restricted areas that did not overlap as well with FA findings (middle cerebellar peduncle, inferior fronto-occipital fasciculus, interior longitudinal fasciculus, and anterior limb of internal capsule) (Table 6 and Fig. 1c,d).

#### Age by group interactions

**Figure 2:**
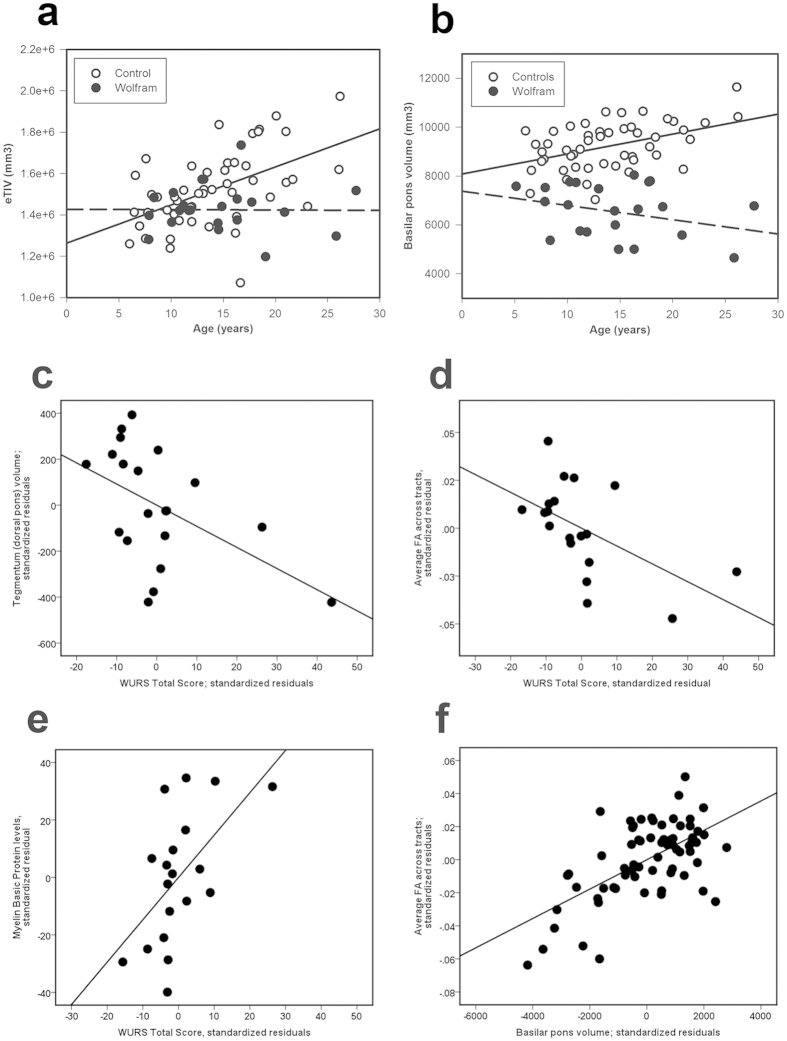
Scatterplots of brain measures with significant age by group interactions, as well as significant brain-behavior and brain-brain relationships within the Wolfram group only. Significant age by group interactions were seen in (**a**) eTIV (F_1,66_ = 7.47, p = 0.008) and (**b**) basilar (ventral) pons (F_1,66_ = 7.65, p = 0.007). Correlations were seen between the WURS Total Score (higher scores are worse) and (**c**) tegmentum (dorsal pons) volume (r_15_ = −0.50, p = 0.040; higher volume is better), (**d**) average FA across tracts (r_15_ = −0.52, p=0.022; higher levels are better), and (**e**) myelin basic protein levels (r_14_ = 0.604, p = 0.013; higher levels are worse), after controlling for age and gender. (**f**) In addition, basilar pons volume correlated with average FA (r_16_ = 0.569, p = 0.014). All brain volumes were also corrected for intracranial volume. Abbreviations: WURS, Wolfram Syndrome Rating Scale; FA, fractional anisotropy.

Brain variables that had an effect of age by group included eTIV (F_1,66_ = 7.47, p = 0.008), Fig. 2a; basilar pons (F_1,66_ = 7.65, p = 0.007), Fig. 2b; uncinate fasciculus RD (F_1,64_ = 5.07, p = 0.028); corpus callosum body FA (F_1,64_ = 5.05, p = 0.028); and corticospinal tract RD (F_1,64_ = 4.02, p = 0.049). However, on inspection of the scatterplots, one older subject was an outlier for all DTI metrics. When this patient’s data was removed, the age by group interactions for DTI measures were no longer significant.

#### Correlations within the Wolfram group

Exploratory correlations between behavioral and brain variables with group differences revealed that DTI parameters were more likely than subcortical regions of interest (e.g. pons) to be associated with behavioral symptoms (see Supplementary Fig. S1). One exception to this was the relationship between tegmentum (dorsal) pons and WURS Total Score (r_15_ = −0.50, p = 0.040), such that lower volume was related to a higher (worse) overall Wolfram syndrome severity score (Fig. 2c). To further explore the role of altered FA, RD and AD in Wolfram syndrome, we computed an average of these parameters across all tracts and correlated these summary variables with key clinical variables. Average FA correlated with WURS total score (r_15_ = −0.52, p = 0.022; Fig. 2d) and WURS Physical score (r_15_ = −0.70, p = 0.002). In addition, myelin basic protein levels correlated with WURS total score (r_14_ = 0.60, p = 0.013; Fig. 2e) and basilar pons volume correlated with average FA (r_16_ = 0.57, p = 0.014; Fig. 2f).

## Discussion

This study provides the most comprehensive and definitive picture of Wolfram syndrome-related brain and behavioral abnormalities to date. The pattern of neuroimaging-derived metrics strongly suggests that reduction in myelin is a primary neuropathological feature of Wolfram syndrome, consistent with existing data from neuronal cell models^17^. We propose that ER stress-related dysfunction may interact with the development of myelin or promote degeneration of myelin during the progression of Wolfram syndrome. If this hypothesis were confirmed in animal or induced pluripotent stem cell (iPS) models, Wolfram syndrome would then fit within a group of neurodevelopmental disorders characterized by ER stress-related impairment of myelination^18^. Lessons learned from the study of this class of disorders may then lead to advances in the treatments for each individual disorder. Our results also highlight regional and early emerging vulnerabilities to Wolfram syndrome. Some of these abnormalities (e.g. myelination markers) appear stable across childhood and early adulthood, suggesting an early developmental failure. Others, such as basilar (ventral) pons, deviate more at older ages from controls, suggesting a degenerative process. Thus, these neuroimaging metrics may provide an objective and quantifiable signature of Wolfram syndrome pathophysiology that will be useful in unraveling the underlying mechanisms of neurological symptoms, focusing the search for biomarkers of change over time and in testing the impact of treatments on the brain.

Our analysis of DTI parameters revealed dramatically reduced FA and increased RD throughout most major white matter tracts. This pattern is recognized as reflecting either demyelination or lack of myelination of axons^40^,^41^. Our previous analysis with a much smaller sample of patients (n = 11) and only convenience controls found similar FA results, but RD was less affected than AD^9^. This current analysis, with its larger sample, age and gender equivalent control groups, and improved analyses (e.g. tractography), provides a more definitive picture and further suggests some clinical significance of decreased myelination in Wolfram syndrome. Greater overall disease severity as indexed by the WURS was related to lower overall FA and greater overall RD (albeit at a trend level). In addition, lower FA and greater RD and AD within specific tracts correlated relatively well with motor-based neurologic measures (e.g. PANESS), supporting the idea that alterations in myelination are related to greater symptom severity. Finally, higher levels of myelin basic protein, an important component of myelin which is known to increase in response to neuronal damage^42^, strongly correlated with overall disease severity in our patients. We have previously shown that cleaved myelin basic protein levels in brain lysates were higher in a mouse model of Wolfram syndrome compared to controls^17^. Thus, myelin basic protein may be a neuropathophysiologically meaningful biomarker of disease severity in Wolfram syndrome. These findings need further exploration within a larger longitudinal sample.

Myelination is one of the most important neurodevelopmental processes that occurs in brain development during childhood and adolescence^43^. Interestingly, myelinating cells (oligodendrocytes) are highly sensitive to ER disruption or compromise due to their need to synthesize a large quantity of myelin membrane proteins, cholesterol, and membrane lipids, placing them at risk of apoptosis^18^. In addition, the ER in mature oligodendrocytes in Wolfram syndrome may be more fragile compared to controls. Thus, ER stress-induced apoptosis of myelinating cells may occur both in the developmental and adult stages of the disease. Recent animal work suggesting increased myelin degradation in a model of Wolfram syndrome^17^ and neuropathological findings of demyelination in a Wolfram patient^19^ support this possibility. ER-stress related effects on myelination are thought to underlie the myelin-specific abnormalities in a number of neurological disorders such as Charcot-Marie-Tooth, Pelizaeus-Merzbacher and Vanishing White Matter Diseases^18^.

Pons volume has been previously noted by our group and others as one of the most obviously affected regions in Wolfram syndrome^2^,^9^,^44^. We advance this literature by showing that this abnormality increases across age, suggesting a degenerative process during childhood and adolescence. In addition, our study determined that basilar (ventral) pons is more affected than tegmentum (dorsal pons) and that volume in this specific region correlates with overall measures of myelination deficits. The basilar pons contains major white matter tracts, such as corticopontine, pontocerebellar and corticospinal fibers, and diffuse and interspersed gray matter known as the pontine nuclei^45^. Importantly, the tegmentum (dorsal pons) is also lower in volume and correlates with overall symptom severity, and there are major regulatory centers that span both of these areas of the pons, including the pontine respiratory group and the reticular formation, which regulates sleep. Sleep apnea and respiratory failure are life-threatening conditions that occur in Wolfram syndrome and deserve further scrutiny in longitudinal analyses. It is possible that the active demyelination of fibers passing through the basilar pons is responsible for decreased volume and the increasing abnormalities with age. The basilar pons normally increases in volume postnatally until early childhood, driven primarily by the addition of new oligodendrocytes and increased myelination^46^. If ER stress dysfunction in Wolfram syndrome is interfering with myelination, this could explain the preferential impact of Wolfram syndrome on the basilar pons. However, we cannot rule out that the gray matter of the basilar pons (e.g. pontine nuclei) is also affected by Wolfram syndrome. Given that dysfunctional myelin can contribute to cell body death^47^, both gray and white matter in the pons could be at risk. Emerging imaging techniques^48^ that may more directly measure myelin in the brain would be helpful in disentangling these possibilities.

Our findings also indicate that structure and function of the visual and auditory systems are related in Wolfram syndrome, but in complex ways. Worse vision was related to more preserved auditory white matter, and worse hearing was related to less preserved visual cortex. Although auditory cortex was thicker in Wolfram patients compared to controls, it did not correlate with visual or auditory function. These complex and somewhat unexpected relationships could be driven in part by false positives, or they could indicate complex compensatory processes to diminishing visual or auditory input. Such a relationship would not be unprecedented in developmental vision and hearing loss conditions^49^, but would require more evidence to support.

Finally, another intriguing finding was that overall, Wolfram patients had smaller skull circumference and intracranial volume and were shorter than controls. It is currently unclear if these differences reflect sampling bias (e.g. our Wolfram families happen to be more petite than control families by chance) or are a result of restrictions in head and body development as has been seen in other genetic neurodegenerative conditions^50^. Interestingly, smaller head size did correlate with worse motor function in the Wolfram group. Similar measures in parents and non-carrier siblings would be necessary to resolve these issues. Importantly, individual brain sizes were taken into account for all of our regional analyses.

This study has some limitations that require discussion. First, our sample size is small compared to studies of more common neurodegenerative diseases. Genotype-phenotype correlations within Wolfram syndrome are of great interest^4^,^6^, but in order to explore these issues for the neuroimaging metrics here, we would need a much larger and more diverse sample. On the other hand, this study is the largest and most comprehensive evaluation of neurological and quantitative neuroimaging abnormalities in this rare condition (1 in 500,000 to 1,000,000)^2^,^51^ to date, and provides a significant insight into its neuropathophysiology. Second, cross-sectional results may not predict longitudinal change. Recognizing this limitation, we have been assessing Wolfram patients and controls annually in order to disentangle neurodegenerative changes from normal brain developmental trajectories; analyses are underway. Third, despite the benefits of *in vivo* neuroimaging, neuropathological examination of patients with Wolfram syndrome or animal models with a clear neurophenotype would provide more definitive cellular level information.

In conclusion, the results of this study have both heuristic and clinical value. Our findings provide important mechanistic clues underlying the regional and tissue-specific neuropathological changes in Wolfram syndrome. Insights into the interaction between the neurodevelopmental process of myelination and the underlying ER stress-related mechanism of cell death in Wolfram syndrome may lead to more targeted brain focused animal studies. In addition, this potential interaction would further highlight possible interplay between neurodevelopment and neurodegeneration, an area of significant interest in other disorders as well^50^. Further studies using animal models and Wolfram syndrome iPS-derived oligodendrocytes exploring these issues will be needed to develop empirically based and innovative treatments for the life-threatening neurodegeneration in Wolfram syndrome. Such studies may lead to the development of novel treatments for other ER stress-associated neurodegenerative diseases. In addition, we propose that markers of myelination and regionally specific brain volumes (e.g. basilar pons) have practical and clinical value as brain biomarkers for clinical trials and natural history studies of Wolfram syndrome.

## Additional Information

**How to cite this article**: Lugar, H. M. *et al*. Neuroimaging evidence of deficient axon myelination in Wolfram syndrome. *Sci. Rep*. **6**, 21167; doi: 10.1038/srep21167 (2016).

## Supplementary Material

Supplementary Information

## Figures and Tables

**Table 1:** Mean and standard deviation of demographic and clinical variables across Wolfram and control groups.

Variable	Wolfram patients (N = 21)		Diabetic controls (N = 24)		Healthy controls (N = 26)		Wolfram vs. All controls	WFS vs. T1DM vs. HC
	Mean	SD	Mean	SD	Mean	SD	p	p
Age (years)	14.33	5.80	13.61	4.68	14.53	5.24	0.859	0.813
Gender (F/M)	13/8		14/10		12/14		0.444	0.513
VIQ (standard score)	112.62	15.14	107.92	15.52	108.85	8.86	0.449	0.747
Parent WTAR (verbal IQ)	104.00	9.68	104.65	9.08	110.05	7.18	0.224	0.056
Mini-BESTest (summary score)	22.05	2.92	25.46	2.13	25.88	1.58	**<0.001**	**<0.001**
Mini-BESTest *TUG* (sec)	7.48	1.48	6.68	1.36	6.29	1.10	**0.004**	**0.007**
Mini-BESTest *DT-TUG* (sec)	9.36	1.82	8.87	3.53	8.06	1.68	0.162	0.216
UPSIT (# correct)	24.48	7.15	30.58	5.63	30.19	4.64	**<0.001**	**<0.001**
HbA1c (%)	7.33	1.19	8.32	1.01	5.28	0.27	0.209	**<0.001**
Diabetes Duration (years)	8.77	4.64	7.86	4.90				0.990*
Pre-MRI Blood Glucose (mg/ml)	189.25	63.69	191.42	55.95				0.905*
Post-MRI Blood Glucose (mg/ml)	152.95	72.76	174.87	66.26				0.313*

P values are from the main effect of group in a univariate analysis. Maximum N values are given per group, but some variables had missing data as reported in the Results section. Abbreviations: WFS, Wolfram syndrome; T1DM, Diabetic controls; HC, healthy controls; SD, standard deviation; VIQ, verbal intelligence quotient; WTAR, Wechsler Test of Adult Reading; Mini-BESTest, mini-Balance Evaluation Systems Test; *TUG*, Timed Get up and Go; *DT-TUG*, Get Up and Go with Dual Task; *UPSIT,* University of Pennsylvania’s Smell Identification Test.

**Table 2:** Mean and standard deviation of clinical variables in Wolfram patients.

Variable	Wolfram patients		
	Mean	SD	N
WURS Total (symptom score)	15.80	14.16	20
WURS Physical (symptom score)	8.80	7.13	20
WURS Behavioral (symptom score)	7.24	9.09	20
PANESS (symptom score)	32.62	11.66	21
Gait Double Support (%)	21.61	3.65	18
Color Vision Score (# correct)	5.52	8.09	21
Visual Acuity (logMAR)	0.69	0.40	21
Retinal Thickness (μm)	59.5	8.70	18
High Frequency Average (dB HL)	34.88	23.19	21
Pure Tone Average (dB HL)	13.89	12.54	21
Speech Intelligibility (%)	80.72	23.46	17
Myelin Basic Protein (ng/ml)	122.38	22.76	20

Maximum N values are given for each measure. Some variables had missing data as reported in the Results section. Abbreviation. SD: standard deviation; WURS: Wolfram United Rating Scale; PANESS: Physical and Neurological Examination for Subtle Signs; HL, hearing level (higher is worse).

**Table 3:** Global brain measures and regional subcortical volumes for Wolfram and control groups.

Brain Measure	Wolfram (N = 20)		All Controls (N = 50)		p
	Mean	SEM	Mean	SEM	
Skull circumference	528.12	3.66	538.56	2.31	**0.019**
Estimated intracranial volume (eTIV)	1426804	30386	1518459	19176	**0.013**
Total cortical gray matter volume	513305	5166	510737	3260	0.676
Total cortical white matter volume	383705	4678	394954	2952	**0.046**
Average surface area	85349	622	83781	392	**0.037**
Average thickness	2.64	0.02	2.68	0.01	0.136
Total subcortical gray matter volume	54952	767	58101	484	**<0.001**
Basilar (ventral) pons volume*	6548	215	9238	139	**<0.001**
Tegmentum (dorsal pons) volume*	2770	70	2966	46	**0.023**
Midbrain volume*	2745	86	2963	55	**0.036**
Medulla volume*	2089	67	2303	82	**0.010**
Cerebellar white matter volume	11964	279	14253	176	**<0.001**
Cerebellar gray matter volume	46279	945	50095	596	**0.001**
Thalamus volume	6501	105	7161	66	**<0.001**
Caudate volume	3950	86	3870	54	0.433
Putamen volume	5630	140	5945	89	0.063
Pallidum volume	1484	42	1678	27	**<0.001**
Hippocampal volume	3868	68	3851	43	0.837
Amygdala volume	1663	35	1569	22	**0.027**
Accumbens volume	653	15	659	10	0.769
Corpus callosum volume	2642	100	2784	63	0.230

P values are from the main effect of group in a univariate analysis, controlling for age and gender. Significant results at the p<0.05 level are in bold. Freesurfer results that survived a Bonferroni correction for multiple comparisons (p = 0.0015) are underlined. *Twenty-one Wolfram patients were included in these analyses. All volumes are corrected by eTIV. Abbreviations: SEM, standard error of the mean.

**Table 4:** A priori cortical measures for Wolfram and control groups.

Brain Measure	Wolfram (N = 20)		All Controls (N = 50)		p
	Mean	SEM	Mean	SEM	
V1 Surface area	2325	55	2520	35	**0.004**
V1 Gray matter volume	4139	113	4707	72	**<0.001**
V1 Thickness	1.72	0.02	1.81	0.01	**<0.001**
V2 Surface area	2692	46	2722	29	0.583
V2 Gray matter volume	5726	136	6104	86	**0.022**
V2 Thickness	2.00	0.02	2.08	0.01	**0.001**
Primary auditory cortex surface area	320	9	298	6	0.057
Primary auditory cortex volume	1087	35	1001	22	**0.040**
Primary auditory cortex thickness	2.68	0.04	2.70	0.02	0.749
Secondary auditory cortex surface area	675	16	594	10	**<0.001**
Secondary auditory cortex volume	2030	55	1870	35	**0.017**
Secondary auditory cortex thickness	2.78	0.02	2.83	0.02	0.135

P values are from the main effect of group in a univariate analysis, controlling for age and gender. Significant results at the p<0.05 level are in bold. Freesurfer results that survived a Bonferroni correction for multiple comparisons (p = 0.0015) are underlined. Units: Volume, mm^3^; surface area, mm^2^; thickness, mm. All volumes and surface areas are corrected by eTIV. Thicknesses are corrected by global thickness. Abbreviations: SEM, standard error of the mean; V1, primary visual cortex; V2, secondary visual cortex.

**Table 5:** DTI parameters for white matter tracts in Wolfram and control groups.

Brain Measure	Wolfram (N = 20)		Controls (N = 48)		p
	Mean	SEM	Mean	SEM	
Uncinate Fasciculus FA	409	6	407	4	0.705
Uncinate Fasciculus RD	590	8	583	5	0.403
Uncinate Fasciculus AD	1144	8	1119	5	**0.008**
Optic Radiation FA	455	7	515	5	**<0.001**
Optic Radiation RD	587	9	497	6	**<0.001**
Optic Radiation AD	1212	10	1158	7	**<0.001**
Middle Cerebellar Peduncle FA	517	10	553	7	**0.005**
Middle Cerebellar Peduncle RD	462	11	412	7	**<0.001**
Middle Cerebellar Peduncle AD	1106	16	1072	10	0.077
Inferior Fronto-Occipital Fasciculus FA	488	6	541	4	**<0.001**
Inferior Fronto-Occipital Fasciculus RD	555	8	487	5	**<0.001**
Inferior Fronto-Occipital Fasciculus AD	1241	11	1227	7	0.285
Arcuate Fasciculus FA	505	5	509	3	0.555
Arcuate Fasciculus RD	471	6	462	4	0.188
Arcuate Fasciculus AD	1077	8	1066	5	0.240
Acoustic Radiation FA	464	7	500	4	**<0.001**
Acoustic Radiation RD	537	8	495	5	**<0.001**
Acoustic Radiation AD	1144	8	1130	5	0.118
Corpus Callosum Body FA	620	5	628	3	0.213
Corpus Callosum Body RD*	437	7	420	5	**0.047**
Corpus Callosum Body AD	1332	9	1312	6	0.081
Corticospinal Tract FA*	657	7	676	5	**0.042**
Corticospinal Tract RD	397	9	376	6	0.062
Corticospinal Tract AD	1345	14	1334	9	0.516

P values are from the main effect of group in a univariate analysis, controlling for age and gender. Significant results at the p < 0.05 level are in bold. Results that survived a Bonferroni correction for multiple comparisons (p = 0.0019) are underlined. *When a borderline outlier was removed, these results were no longer significant. Units: FA, 10^-3^ mm^2^/s; RD and AD, 10^-6 ^mm^2^/s. Abbreviations: DTI, diffusion tensor imaging; SEM, standard error of the mean; FA, fractional anisotropy; RD, radial diffusivity; AD, axial diffusivity.

**Table 6:** TBSS analyses and results comparing Wolfram and control groups after multiple comparison correction.

Measure	Contrast	p < 0.05	White matter regions
FA	All controls > Wolfram	Yes	Parts of corpus callosum body
			Corticospinal tract
			Inferior fronto-occipital fasciculus
			Optic radiations
	Wolfram > All controls	No	
RD	Wolfram > All controls	Yes	Middle cerebellar peduncle
			Corticospinal tract
			Interior longitudinal fasciculus
			Inferior fronto-occipital fasciculus
			Optic radiations
			Superior longitudinal fascisulus
	All controls > Wolfram	No	
AD	Wolfram > All controls	Yes	Middle cerebellar peduncle
			Inferior fronto-occipital fasciculus
			Interior longitudinal fasciculus
			Anterior limb of internal capsule
	All controls > Wolfram	No	

Abbreviations: TBSS, tract-based spatial statistics; FA, fractional anisotropy; RD, radial diffusivity; AD, axial diffusivity.
