# Supplementary material for: Neuroimaging evidence of deficient axon myelination in Wolfram syndrome
Source: Sci Rep. 2016 Feb 18;6:21167. doi: 10.1038/srep21167 (PMC4758056; doi:10.1038/srep21167)
Supplement: Supplementary Information [file srep21167-s1.pdf]

Neuroimaging evidence of deficient axon myelination in Wolfram syndrome

Heather M. Lugar, M.A.[1], Jonathan M. Koller, BSBME, BSEE[1], Jerrel Rutlin, B.S[1], Bess A. Marshall, M.D.[4,6], Kohsuke Kanekura, M.D., Ph.D.[5], Fumihiko Urano, M.D., Ph.D.[5], Allison Bischoff, BA[1], Joshua S. Shimony, M.D., Ph.D.[3], *Tamara Hershey Ph.D[1,2,3] and the Washington University Wolfram Syndrome Study Group

Departments of Psychiatry[1], Neurology[2], Radiology[3], Pediatrics[4], Medicine[5], Washington University School of Medicine and St. Louis Children's Hospital[6], St. Louis, MO 63110, USA

***Corresponding Author:**

Tamara Hershey, Ph.D.

Campus Box 8134

4525 Scott Avenue

Washington University School of Medicine

St. Louis, Missouri 63110

Phone: (314) 362-5593

Fax: (314) 362-0168

Email: tammy@npg.wustl.edu

Supplementary Figure S1. **Correlations between brain and behavioral measures within the Wolfram group.** Within the Wolfram group, significant Pearson's correlations coefficients are shown between brain and behavioral measures which had significant group effects or were abnormal compared to clinical norms. Significance was set at $p < 0.05$, after controlling for age and gender. Abbreviations: eTIV, estimated intracranial volume; FA, fractional anisotropy; RD, radial diffusivity; AD, axial diffusivity; WURS, Wolfram Syndrome Rating Scale; PANESS, Physical and Neurological Examination for Subtle Signs; Mini-BESTest, mini-Balance Evaluation Systems Test; TUG, Timed Get Up and Go; UPSIT, University of Pennsylvania's Smell Identification Test. *Behavioral measures in which higher scores are better.

	eTIV	Cortical White Matter Volume	Average Surface Area	Subcortical Gray Matter Volume	Basilar (Ventral) Pons Volume	Tegmentum (Dorsal Pons) Volume	Midbrain Volume	Medulla Volume	Cerebellar White Matter Volume	Cerebellar Gray Matter Volume	Thalamus Volume	Pallidum Volume	Amygdala Volume	V1 Surface Area	V1 Gray Matter Volume	V1 Thickness	V2 Gray Matter Volume	V2 Thickness
* Total Color Vision Score					.57				.54									
* Visual Acuity																		
* Retinal Thickness															.54			
Audio Puretone											-.57							
Audio High Frequency											-.74					.62		
* Audio Speech Intelligibilty											.68					-.55		
WURS Total						-.56												
WURS Physical Total	-.50																	
WURS Behavioral Total																		
Total PANESS	-.58	-.56																
Gait Double Support									.72	.59						.62		
* Mini-BESTest Summary Score	.52																	
Mini-BESTest *TUG*																		
* UPSIT								.54					.48					

	Primary Auditory Gray Matter Volume	Secondary Auditory Cortex Surface Area	Secondary Auditory Gray Matter Volume	Uncinate Fasciculus AD	Optic Radiation FA	Optic Radiation RD	Optic Radiation AD	Middle Cerebellar Peduncle FA	Middle Cerebellar Peduncle RD	Inferior Fronto-Occipital Fasciculus FA	Inferior Fronto-Occipital Fasciculus RD	Acoustic Radiation FA	Acoustic Radiation RD	Corpus Callosum Body RD	Corticospinal Tract FA	Average FA	Average RD	Average AD
* Total Color Vision Score												.62	-.56					
* Visual Acuity												-.79	.71					
* Retinal Thickness																		
Audio Puretone																		
Audio High Frequency																		
* Audio Speech Intelligibilty										.56				-.62				
WURS Total																-.52		
WURS Physical Total					-.65					-.73		-.52				-.70		
WURS Behavioral Total																		
Total PANESS					-.58					-.71	.57			.57				
Gait Double Support			-.48						-.63									
* Mini-BESTest Summary Score																		
Mini-BESTest *TUG*				-.54														
* UPSIT				-.52			-.51											

Supplementary Table S1. Study age (SA) and age at diagnosis (in years) of optic atrophy (OA), diabetes mellitus (DM), diabetes insipidus (DI), and hearing loss (HL) in each Wolfram patient, as well as their genetic mutations. #, unknown. Superscripts a, b, and c represent sets of siblings from four different families; fifteen families, in total, participated. *Patients who have data from a different time point represented in Hershey et al., 2012.

Patient	SA	OA	DM	DI	HL	Allele 1	Allele 2
*WOLF02	14	9	6	7	NA	c.2648del4; p.F883fs	None identified
*WOLF03	20	6	5	6	6	c.1230_1233del; p.Val412Serfs*29	c.1243_1245del; p.Val415del
*WOLF07	10	7	2	7	NA	c.2002C>T; p.Gln668*	c.2002C>T; p.Gln668*
*WOLF09[a]	16	11	10	14	NA	c.376G>A; p.Ala126Thr	c.1838G>A; p.Trp613*
*WOLF10[a]	14	8	7	11	NA	c.376G>A; p.Ala126Thr	c.1838G>A; p. Trp613*
*WOLF11[a]	11	7	7	8	9	c.376G>A; p.Ala126Thr	c.1838G>A; p. Trp613*
*WOLF12	25	17	7	17	7	c.320G>A; p.Gly107Glu	c.1885C>T; p.Arg629Trp
*WOLF13	8	5	5	7	NA	c.599delT; p.Leu200Argfs*87	c.2254G>T; pGlu752*
*WOLF14	14	7	6	11	10	c.817G>T; p.Glu273*	c.1839G>A; p.Trp613*
WOLF15	11	7	3	10	9	c.439delC; Arg147fs*163	c. 1620G>A; pTrp540*

*WOLF16	27	13	13	14	NA	c.1240_1242del; p.Phe414del	c.1689_1694del; p.Phe564del;p.Leu565del
*WOLF17[b]	19	15	5	NA	15	c.599T>C; p. Leu200Pro	c.695G>C, p.Arg232Pro
WOLF18	12	10	5	10	NA	c.1251_1252delinsG; p.Phe417Leufs*25	c.1885C>T; p.Arg629Trp
WOLF22	16	12	14	NA	NA	c.605A>G; p.Glu202Gly	c.631G>A; p.Asp211Asn
WOLF23[c]	17	17	5	NA	17	c.739_740del, p.Phe247fs*5	c.1243_1245del, p.Val415del
WOLF24[c]	16	10	4	5	14	c.739_740del, p.Phe247fs*5	c.1243_1245del, p.Val415del
WOLF25[c]	7	#	5	NA	NA	c.739_740del, p.Phe247fs*5	c.1243_1245del, p.Val415del
WOLF27[d]	10	8	3	9	NA	c.1230_1233del; p.Val412Serfs*29	c.1243_1245del, p.Val415del
WOLF28[d]	7	5	5	NA	NA	c.1230_1233del: p.Val412Serfs*29	c.1243_1245del, p.Val415del
WOLF29[b]	5	#	NA	NA	3	c.599T>C, p.Leu200Pro	c.695G>C, p.Arg232Pro
WOLF31	10	7	5	NA	10	c.2140_2163dup24 p.Asn714_Asn721dup	c.2140_2163dup24 p.Asn714_Asn721dup
